# Deciphering the impacts of modulating the Wnt-planar cell polarity (PCP) pathway on alveolar repair

**DOI:** 10.3389/fcell.2024.1349312

**Published:** 2024-02-27

**Authors:** Sally Yunsun Kim, David McTeague, Sek-Shir Cheong, Matthew Hind, Charlotte H. Dean

**Affiliations:** ^1^ National Heart and Lung Institute, Faculty of Medicine, Imperial College London, London, United Kingdom; ^2^ Royal Brompton and Harefield Hospitals, Guy’s and St Thomas’ NHS Foundation Trust, London, United Kingdom

**Keywords:** Wnt, planar cell polarity (PCP), precision-cut lung slices (PCLS), repair, progenitor, lung, alveolar

## Abstract

Many adult lung diseases involve dysregulated lung repair. Deciphering the molecular and cellular mechanisms that govern intrinsic lung repair is essential to develop new treatments to repair/regenerate the lungs. Aberrant Wnt signalling is associated with lung diseases including emphysema, idiopathic pulmonary fibrosis and pulmonary arterial hypertension but how Wnt signalling contributes to these diseases is still unclear. There are several alternative pathways that can be stimulated upon Wnt ligand binding, one of these is the Planar Cell Polarity (PCP) pathway which induces actin cytoskeleton remodelling. Wnt5a is known to stimulate the PCP pathway and this ligand is of particular interest in regenerative lung biology because of its association with lung diseases and its role in the alveolar stem cell niche. To decipher the cellular mechanisms through which Wnt5a and the PCP pathway affect alveolar repair we utilised a 3-D *ex-vivo* model of lung injury and repair, the AIR model. Our results show that Wnt5a specifically enhances the alveolar epithelial progenitor cell population following injury and surprisingly, this function is attenuated but not abolished in *Looptail (Lp)* mouse lungs in which the PCP pathway is dysfunctional. However, *Lp* tracheal epithelial cells show reduced stiffness and *Lp* alveolar epithelial cells are less migratory than wildtype (WT), indicating that *Lp* lung epithelial cells have a reduced capacity for repair. These findings provide important mechanistic insight into how Wnt5a and the PCP pathway contribute to lung repair and indicate that these components of Wnt signalling may be viable targets for the development of pro-repair treatments.

## Introduction

The lungs have an innate ability for repair facilitated by facultative stem/progenitor cell populations and additional repair processes such as cell migration and spreading (Crosby and Waters, 2010; Hogan et al., 2014; Alysandratos et al., 2021). This capacity for repair is vital to maintain healthy lungs since they are frequently exposed to injurious substances including diesel, smoke, allergens or pathogenic agents. However, some individuals are unable to mount or maintain an effective repair response resulting in acute or chronic lung diseases (Dean and Lloyd, 2017). Understanding the cellular and molecular mechanisms driving intrinsic lung repair is important for the development of regenerative medicine-based approaches to treat diseases where repair is dysfunctional (Lau et al., 2012; Parekh et al., 2020).

One family of growth factors that is frequently associated with regeneration and tissue repair is the Wingless-type MMTV integration site family (Wnts). There are 19 mammalian Wnt ligands that govern a diverse array of cellular processes at all life stages (Niehrs, 2012; Wiese et al., 2018). Mutations in Wnts and Wnt signalling pathway genes underly a variety of congenital and adult diseases ranging from split hand foot syndrome, Osteogenesis imperfecta to type II diabetes and lung diseases such as Idiopathic pulmonary fibrosis (IPF) and pulmonary arterial hypertension (PAH) (Kanazawa et al., 2004; Baron and Kneissel, 2013; Skronska-Wasek et al., 2018).

Wnt signalling is complex and individual Wnt ligands can stimulate several downstream signalling cascades: the canonical pathway for which β-catenin is central and two non-canonical pathways, the calcium pathway and planar cell polarity (PCP) pathway (Nusse, 2012).

At one time, individual Wnt ligands were believed to only stimulate either the canonical pathway or non-canonical pathways but it is now known that many Wnt ligands are capable of stimulating any of the downstream cascades, depending on the particular temporal and cellular environment (Rao and Kuhl, 2010). Despite their pleiotropic potential, some Wnt ligands do exhibit a strong bias for signalling via one particular pathway. An example of this is Wnt5a which is primarily known to signal via the non-canonical PCP pathway (Rogers and Scholpp, 2022).

Work carried out across various model organisms has identified a group of six core PCP proteins as well as a growing number of potential co-receptors e.g. Ror2 and effectors such as Fuzzy or Inturned (Adler and Wallingford, 2017). One of the most well studied core PCP genes is Van Gogh (Vang). In mammals there are two homologues: Vangl Planar Cell Polarity Proteins, 1 and 2 (Vangl1 and Vangl2). Looptail mice carry a missense mutation in *Vangl2* which not only results in loss of Vangl2 function but also effects additional PCP pathway components, including Vangl1 (Montcouquiol et al., 2006; Yin et al., 2012; Pryor et al., 2014), because of the disruption to multiple pathway components, Looptail mice have been used extensively as a tool with which to study PCP pathway function.

The Wnt stimulated PCP pathway (Wnt-PCP) induces actin cytoskeleton rearrangements and through this role, the pathway is required for planar orientation of cells, tissue morphogenesis and directed cell migration during development of multiple glands and organs (Li et al., 2005; Davey and Moens, 2017; Henderson et al., 2018; Li et al., 2022).

In the lungs, the Wnt-PCP pathway is necessary to form and shape the airways and alveoli via modulating actin cytoskeleton mediated functions, particularly, cell migration (Yates et al., 2010; Poobalasingam et al., 2017; Cheong et al., 2020; Vladar and Konigshoff, 2020). Building on these findings, two separate investigations employing mice carrying floxed alleles of *Wnt5a* and the PCP pathway genes *Ror2* and *Vangl2* found common alveologenesis defects including alveolar insufficiency and abnormal cytoskeleton organisation (Li et al., 2020; Zhang et al., 2020).

Beyond organ development, Wnt5a-PCP signalling is also important for adult lung homeostasis and repair (Baarsma et al., 2017; Poobalasingam et al., 2017). In emphysema patient lungs, where the alveoli are damaged by repeated injury, transcript levels of Wnt5a and PCP pathway genes *Vangl2* and *Scrib* are significantly reduced (Poobalasingam et al., 2017; Zhang et al., 2020).

Multiple stem cell/progenitor cell populations have been identified in the lungs which reside at different locations during homeostasis (Wu and Tang, 2021). As well as resident alveolar progenitor cells that are derived from Alveolar type II cells (ATII), reports have shown that a different population of progenitor cells can migrate from the bronchioalveolar ducts and distal airways into the alveoli upon lung injury. The tissue resident alveolar progenitor cells are a set of facultative ATII cells that respond to injury, these alveolar epithelial progenitor (AEP) cells can be identified by the presence of pro-surfactant protein C (proSP-C) (Barkauskas et al., 2013) and also by the cell surface marker transmembrane 4 L six family member 1 (TM4SF1) (Zacharias et al., 2018).

Both Wnt5a and PCP pathway genes have been shown to play a role in lung regeneration and/or repair (Poobalasingam et al., 2017; Jain et al., 2022; Li et al., 2022). Nabhan et al. discovered that Wnt5a secreted from fibroblasts activates a population of alveolar type II progenitor cells (ATII) to help drive repair following influenza-induced lung injury (Nabhan et al., 2018) and the addition of Wnt5a leads to accelerated repair of the alveolar epithelial cell line-A549s whereas depletion or loss of Vangl2 function reduces the ability of A549s or primary tracheal epithelial cells to repair following a scratch wound (Poobalasingam et al., 2017; Cheong et al., 2020).

However, given the ability of Wnt ligands to stimulate several different intracellular pathways, it is essential to decipher the precise effects of both Wnt5a itself and the PCP pathway on lung repair and regeneration to enable the development of a successful treatment with limited detrimental side effects.

Using a recently developed 3D model of lung injury and repair in precision-cut lung slices (PCLS), the Acid-Injury and Repair (AIR) model, we investigated the cell-specific effects of Wnt5a and PCP pathway dysfunction in adult lung homeostasis and in the early stages of tissue repair after injury. Here we show that Wnt5a specifically potentiates the induction of AEP cells following injury in wildtype (WT) lung tissue. In *Vangl2* ^*Lp/+*^ lungs, in which the PCP pathway is disrupted, the pro-repair capacity of Wnt5a is attenuated but remains significantly increased.

## Materials and methods

### Mice

All animal maintenance and procedures were conducted in compliance with the Animal (Scientific Procedures) Act 1986 and approved by the South Kensington AWERB committee at Imperial College London. Mice were housed in specific pathogen-free conditions and given food and water *ad libitum*. *Looptail (Lp)* mice were genotyped by MRC Harwell (Oxford, United Kingdom) using a pyrosequencing assay. *Vangl2* ^*Lp*^ mice carry *a* point mutation, S464N, that results in loss of Vangl2 function (Kibar et al., 2001; Murdoch et al., 2001). The following primer sequences were used: Lp_SNP1_For_BiotinGTCCTGGCGCTTCAAGAGGA, Lp_SNP1_RevNNNGGCCAAACAGTGGACCTTGG, Lp_SNP1_SeqRCAGTGGACCTTGGTGA. Cycling conditions were as follows: Step 1 95°C ^o^ 5 min followed by 44 cycles of Step 2 95°C 15 s, Step 3 60 C 30 s, Step 4 72°^o^C 15 s and a final step of 72°C 5 min *Vangl2* ^*Lp*^ mice were maintained on a C3H/HeH background. Male and female adult *Vangl2* ^*Lp/+*^ mice and Wildtype (WT) littermates (8–12 weeks old) were humanely killed using intraperitoneal injection of pentobarbital before harvesting lungs.

### Generation of precision-cut lung slices and acid injury and repair (AIR) model

Lungs were harvested from WT and *Vangl2* ^*Lp/+*^ mice and precision-cut lung slices (PCLS) were generated as described previously (Akram et al., 2019). Briefly, after exposing the trachea and chest, lungs were harvested after filling with low melting point agarose 2% solution and PCLS were generated at 300 μm thickness using an automated vibratome (Compresstome^®^ VF-300-0Z; Precisionary Instruments LLC). To compare tissue repair responses in WT and *Vangl2* ^*Lp/+*^ mice, we adopted the AIR model where a spatially restricted area of PCLS was injured by brief application of 0.1 M hydrochloric acid. PCLS were subsequently cultured for 48h in DMEM (Life Technologies, Cat. no. 10569010) with 1% penicillin-streptomycin at 37°C and 5% CO_2_ before immunostaining for relevant markers of repair, as previously described (Kim et al., 2020). For gene expression assays, acid injury was applied to whole PCLS for 1 min and slices were then cultured for 48h prior to RNA extraction.

### Wnt5a and Box5 treatment of PCLS

To investigate the pro-repair effects of Wnt5a, WT and *Vangl2* ^*Lp/+*^ AIR-PCLS were treated with recombinant human/mouse Wnt5a (Bio-Techne Ltd., Cat. no. 645-WN-010) at a pre-optimised concentration of 1 μg/mL, immediately following acid-injury. Some PCLS were pre-treated for 40 min with the Wnt5a antagonist, Box5 (Merck Millipore, Cat. no. 681673) at a pre-optimised concentration of 100 µM and then incubated with both Wnt5a 1 μg/mL + Box5 100 µM for 48h to test whether effects observed upon Wnt5a treatment were specific to this ligand. Control, uninjured PCLS were also treated with Wnt5a 1 μg/mL or Wnt5a 1 μg/mL + Box5 100 µM for comparison.

### Immunofluorescence staining of PCLS

Immunofluorescence staining of PCLS was conducted as described previously (Kim et al., 2020). Briefly, PCLS were fixed using 4% paraformaldehyde at RT for 15 min, washed three times with PBS, permeabilised with 0.5% Triton X-100 for 30 min at RT and then incubated with 1% BSA and 0.2% Triton X-100 in PBS (PBS-BT) for 1 h to block non-specific binding. Primary antibodies were diluted in PBS-BT as follows: Anti-mouse and rat Ki67 (eBioscience; Cat. No. 14-5698-82; Clone SolA15) and rabbit anti-prosurfactant protein-C (proSP-C; Millipore; Cat. No. AB3786) 1:500, sheep anti-transmembrane 4 L6 family member 1 (TM4SF1; R&D Systems, Cat. No. AF7514) 1:40, hamster anti-podoplanin (Developmental Studies Hybridoma Bank; Cat. No. 8.1.1) 1:50. All primary antibodies were incubated overnight at 4°C then washed three times with PBS-BT. PCLS were incubated with secondary antibodies for 2 h at RT in the dark, using goat anti-rat IgG Alexa Fluor 568 (Invitrogen; Cat. No. A-11077) at 1:500 for Ki67, goat anti-rabbit IgG Alexa Fluor 488 (Invitrogen; Cat. No. A-11008) at 1:500 for proSP-C, donkey anti-sheep IgG Alexa Fluor 594 (Invitrogen; Cat. No. A-11016) at 1:250 for TM4SF1 and goat anti-hamster IgG Alexa Fluor 568 (Invitrogen; Cat. No. A-21112) at 1:250 for podoplanin. After washing three times with PBS-BT, PCLS were incubated with DAPI 2 μg/mL for 15 min at RT in the dark, washed once then mounted on glass slides with ProLong^®^ Gold Antifade Mountant.

For vimentin, a live cell imaging probe BioTracker™ TiY Vimentin Live Cell Dye (Merck Millipore, Cat. no. SCT059) was used at a concentration of 5 μM, diluted in DMEM and applied to PCLS for 1 h at 37°C and 5% CO_2_. Vimentin-stained PCLS were fixed using 4% paraformaldehyde at RT for 15 min, washed three times with PBS then counterstained with DAPI, washed once and mounted for imaging.

### Fluorescence microscopy and quantification

For widefield microscopy, immunostained PCLS were imaged using a Zeiss Axio Observer widefield microscope using × 40 air objective with ApoTome and Zen software. Images were acquired from three separate fields of view per injured or uninjured region of each AIR-PCLS and from each control, uninjured PCLS. Quantification was conducted using an ImageJ macro that was specifically designed for counting fluorescently labelled cells on PCLS or manual counting (Kim et al., 2020). Confocal microscopy was conducted using a Leica SP8 inverted confocal microscope with a × 40 oil immersion objective and LAS X software. Quantification of Ki67, proSP-C, TM4SF1 and podoplanin markers was carried out as described above. Quantification of vimentin was calculated using a macro designed to measure the vimentin-positive area as a percentage of whole tissue area. A dot on the graphs represents the mean value obtained from each individual PCLS. Four to five mice per genotype and/or treatment group were used, n numbers (biological replicates) are indicated in the figure legends.

### Gene expression assays

WT and *Vangl2* ^*Lp/+*^ acid-injured or control, uninjured PCLS were cultured for 48 h, then ribonucleic acid (RNA) was isolated using lysing matrix D ceramic beads (MP Biomedicals; Cat. No. 74104) and RNeasy Mini kit (Qiagen; Cat. No. 74104), as described previously (Kim et al., 2020). The concentration and quality of RNA were quantified using High Sensitivity RNA ScreenTape (Agilent Technologies, Cat. no. 5067–5579) and High Sensitivity RNA ScreenTape Sample Buffer (Agilent Technologies, Cat. no. 5067–5580), according to the manufacturer’s instructions. For complementary deoxyribonucleic acid (cDNA) synthesis, 1 µg RNA was converted using a High-Capacity cDNA reverse transcription kit (Applied Biosystems; Cat. no. 4368814). Quantitative real-time polymerase chain reaction (qPCR) was conducted using TaqMan^®^ Fast Advanced Master Mix (Thermo Fisher Scientific; Cat. no. 4444558) and TaqMan^®^ primers (Life Technologies) targeting *Sftpc* (Mm00488144_m1), *Tm4sf1* (Mm00447009_m1), *Axin2* (Mm00443610_m1) and *Ctnnb1* (Mm00483039_m1) and StepOnePlus™ RT-qPCR machine (Applied Biosystems). Relative levels of gene expression were calculated after normalising gene levels to the housekeeping gene *Gusb* (Mm01197698_m1). To determine the changes in *Vangl2* ^*Lp/+*^ PCLS compared to WT PCLS, gene expression fold change was calculated and the values for the *Vangl2* ^*Lp/+*^ PCLS were then normalised to WT PCLS data.

### Isolation of alveolar epithelial cells from WT and *Vangl2* ^*Lp/+*^ mouse lungs

Alveolar epithelial cells from WT and *Vangl2* ^*Lp/+*^ mice were obtained using the method described in Cheong et al. (Cheong et al., 2020). After exposing the trachea and chest of mice, a small insertion was made to the trachea to insert a cannula and lungs were filled with 1 mL Dispase 50 U/mL solution (Corning, Cat. no. 354235) followed by 200 µL agarose 1% (Cat. no. A9414) dissolved in Dulbecco’s Modified Eagle Medium (DMEM; Life Technologies, Cat. no. 10569010) then ice was placed on top of the lung/trachea for agarose to solidify. Lungs were harvested and incubated in Dispase solution at room temperature for 45 min. Lung lobes were separated from the trachea and main airways; lobes were then homogenised using forceps in 3 mL of DMEM containing 25 mM 4-(2-hydroxyethyl)-1-piperazineethanesulfonic acid (HEPES; Life Technologies, Cat. no. 15630-122), 1% penicillin and streptomycin (Life Technologies, Cat. no. 15140122) and 50   U/mL DNase I (Sigma-Aldrich, Cat. no. DN25). The homogenised mixture was passed through 100 μm, 70 μm and 30 μm cell strainers then cell suspension was centrifuged for 5 min at 550 × *g* at 4°C to pellet cells with a thin layer of residual agarose above the pellet, which was removed without disturbing the cells. Cells were resuspended in 1 mL DMEM/HEPES/DNase medium and primary alveolar epithelial cells were isolated using CD326 (EpCAM) MicroBeads (Miltenyi Biotec, Cat no. 130-105-958) as per the manufacturer’s protocol. Briefly, the number of total cells were counted to determine the number of beads to be used. Cells were centrifuged for 5 min at 550 × *g* at 4°C then resuspended in MACS buffer (bovine serum albumin 0.5% and EDTA 2 mM in PBS) containing CD31 and CD45 MicroBeads (Miltenyi Biotec, Cat no. 130-097-418 and 130-052-301, respectively) to deplete endothelial cells and leukocytes by magnetic bead separation using a pre-washed LS column on a MACS Separator. The number of cells in the effluent was determined and CD326 (EpCAM) MicroBeads were added to the cell suspension then incubated at 4°C for 20 min. Cells were pelleted and resuspended in MACS buffer then EpCAM-positive epithelial cell suspension was collected by magnetic bead separation.

### Immunofluorescence staining and imaging of cells

Primary cells extracted from alveolar or tracheal lung regions were fixed in 4% paraformaldehyde for 10 min, washed three times using PBS then incubated in blocking solution with a permeabilising agent (0.2% Triton X-100; Sigma-Aldrich, Cat. no. RES9690T-A101%X and 1% bovine serum albumin, BSA; Sigma-Aldrich, Cat. no. A7030) for 45 min. Cells were incubated with a combination of pan-cytokeratin (Sigma-Aldrich, Cat. no. C2931) and vimentin (Biorbyt, Cat. no. orb304659) antibodies, both diluted at 1:200 or with a combination of proSp-C at 1:500 (proSP-C; Millipore; Cat. No. AB3786) and podoplanin at 1:75 (Developmental Studies Hybridoma Bank; Cat. No. 8.1.1) in 0.2% Triton-X and 1% BSA for 2 h at room temperature (RT) with gentle rocking. After washing three times with PBS, cells were incubated with secondary antibodies: either, donkey anti-mouse IgG, Alexa Fluor 594 (Life Technologies, Cat. no. A21203) or goat anti-hamster IgG Alexa Fluor 568 (Invitrogen; Cat. No. A-21112) for podoplanin and goat anti-rabbit IgG, Alexa Fluor 488 (Life Technologies, Cat. no. A11008) at 1:500 for 45 min at RT and washed three times with PBS. Cells were then incubated with DAPI (2 μg/mL) for 10 min to label all nuclei, washed once and mounted onto glass slides using ProLong^®^ Gold (Life Technologies, Cat. no. P36930). Cells were imaged using a Leica SP8 inverted confocal microscope using × 63 oil immersion objective and LAS X software.

### Migration assay of alveolar epithelial cells using transwell inserts

EpCAM-positive WT and *Vangl2* ^*Lp/+*^ alveolar epithelial cells were resuspended in DMEM/F-12 medium (Life Technologies, Cat. no. 11330032) containing 15 mM HEPES, 4 mM glutamine (Invitrogen, Cat. no. 25030-024), 0.03% NaHCO_3_, 0.25 μg/mL Amphotericin B (Gibco, Cat. no. 15290018), 10 U penicillin-streptomycin, 1 × Insulin-Transferrin-Selenium (ITS; Life Technologies, Cat. no. 41400045), 0.1 μg/mL Cholera toxin (Sigma–Aldrich, Cat. no. C8052), 25 ng/mL epidermal growth factor (EGF; Sigma–Aldrich, Cat. no. E4127), 30 μg/mL bovine pituitary extract (BPE; Gibco, Cat. no. 13028014), and 5% fetal bovine serum (FBS; Gibco, Cat. no. 10500064). Cells were seeded onto collagen-coated 8-well chamber slides and double immunostained for either pan-cytokeratin and vimentin or proSP-C and podoplanin using the protocol detailed above, to confirm the phenotype of the isolated cells. For the migration assay, cells were seeded onto a Transwell^®^ insert with 8 μm pores (Corning Incorporated, Cat. no. 3422) to allow migration of cells through the pores. Both top and bottom sides of Transwell inserts were coated with Matrigel and 100,000 cells were seeded per Transwell insert into the top chamber. A concentration gradient was formed using 2.5% FBS in the top chamber and 10% FBS in the bottom chamber. Media inthe bottom chamber was additionally supplemented with 400 ng/mL fibroblast growth factor-10 (FGF10). Cells were allowed to migrate for 48 h at 37°C and 5% CO_2_, then fixed using 4% paraformaldehyde and stained using crystal violet 2% solution (Sigma-Aldrich, Cat. no. HT901-8FOZ) for 30 min. Any remaining cells in the top chamber were carefully removed using a cotton bud then the cells that had migrated to the bottom chamber were imaged after mounting the membrane onto glass slides. The number of migrated cells were determined by adding the number of cells counted in 5 fields of view for each Transwell. Cells from 3 separate transwells per genotype, per experiment were quantified and plotted as individual data points. Four replicate experiments were performed, n = 4.

### Isolation of epithelial cells from WT and *Vangl2* ^*Lp/+*^ mouse trachea

Mouse tracheal epithelial cells were isolated as previously described (Cheong et al., 2020). Tracheas were placed in cold Ham’s F-12 media (Life Technologies, Cat. no. 11765047), connective tissues were removed using forceps then each trachea was cut lengthwise to expose the lumen before incubation in 3 mL of serum-free Ham’s F-12 media containing 0.15% pronase (Roche, Cat. no. 10165921001) overnight at 4°C. Trachea-containing tubes were inverted to dissociate cells from tracheas then 3 mL of Ham’s F-12 containing 20% FBS was added, and tubes were inverted again. Tracheas were then washed three times with fresh Ham’s F-12 containing 10% FBS and all supernatants were combined and centrifuged at 390 *g* at 4°C for 10 min to pellet the cells. Cells were resuspended in 0.5 mg/mL DNase I solution (Sigma-Aldrich, Cat. no. DN25) dissolved in serum-free Ham’s F-12 medium and incubated on ice for 5 min. Cells were then pelleted by centrifugation at 390 *g* at 4°C for 5 min and resuspended in TEC medium consisting of DMEM/F-12 medium (Life Technologies, Cat. no. 11330032) with 15 mM HEPES, 4 mM glutamine, 0.03% NaHCO_3_, 0.25 μg/mL Amphotericin B, 10 U penicillin-streptomycin and 10% FBS. Cells were seeded onto a 6-well plate and incubated at 37°C and 5% CO_2_ for 4 h to allow attachment of fibroblasts to the plastic. The supernatant containing epithelial cells was carefully removed, centrifuged at 390 *g* and 4°C for 5 min and resuspended in TEC medium supplemented with 1 × ITS, 0.1 μg/mL Cholera toxin, 25 ng/mL EGF, 30 μg/mL BPE, 5% FBS, and 0.1 μM retinoic acid (Sigma-Aldrich, Cat. no. R2625). Cells were counted and seeded onto collagen-coated 8-well chamber slides and immunostained for pan-cytokeratin and vimentin to confirm successful isolation of epithelial cells.

### Atomic force microscopy to measure cell stiffness

WT and *Vangl2* ^*Lp/+*^ tracheal epithelial cells were seeded onto a glass bottom dish (Mattek, Cat. no. P35G-1.5–14-C) coated with collagen. At 72 h after seeding, cells were taken to NanoWizard 4 (JPK Bruker, Berlin, Germany) to measure stiffness of cells. Cone-tipped cantilevers (Bruker, PFQNM-LC-A-CAL) with spring constant 0.109 and sensitivity 10.42 nm/V were used at setpoint 0.2 nN for scanning using the Qi™ mode, with scan size ranging 5–30 μm with 256 × 256 pixels. Using JPK Data Processing software, Young’s modulus micrographs were generated and the stiffness of WT and *Vangl2* ^*Lp/+*^ tracheal epithelial cells were compared. A total of eight to ten 0.55–1 μm^2^ areas in each cell periphery were averaged for each micrograph and data from 3-5 micrographs were averaged for each replicate experiment.

### Statistical analysis

All quantified data were analysed using GraphPad Prism version 9.2. Each dot in the graphs represents the mean value obtained from an individual precision-cut lung slice. Data is presented as the mean of all the biological replicates with standard error of the mean (SEM). For statistical tests, Mann Whitney-U test was used for datasets comparing two groups, Kruskal–Wallis test was used for datasets comparing three groups and a Two-way ANOVA was used for multiple comparisons between untreated and Wnt5a/Box5 treatments for injured and uninjured PCLS.

## Results

### Planar cell polarity (PCP) pathway dysfunction does not affect alveolar epithelial cell homeostasis in adult lungs

**FIGURE 1:**
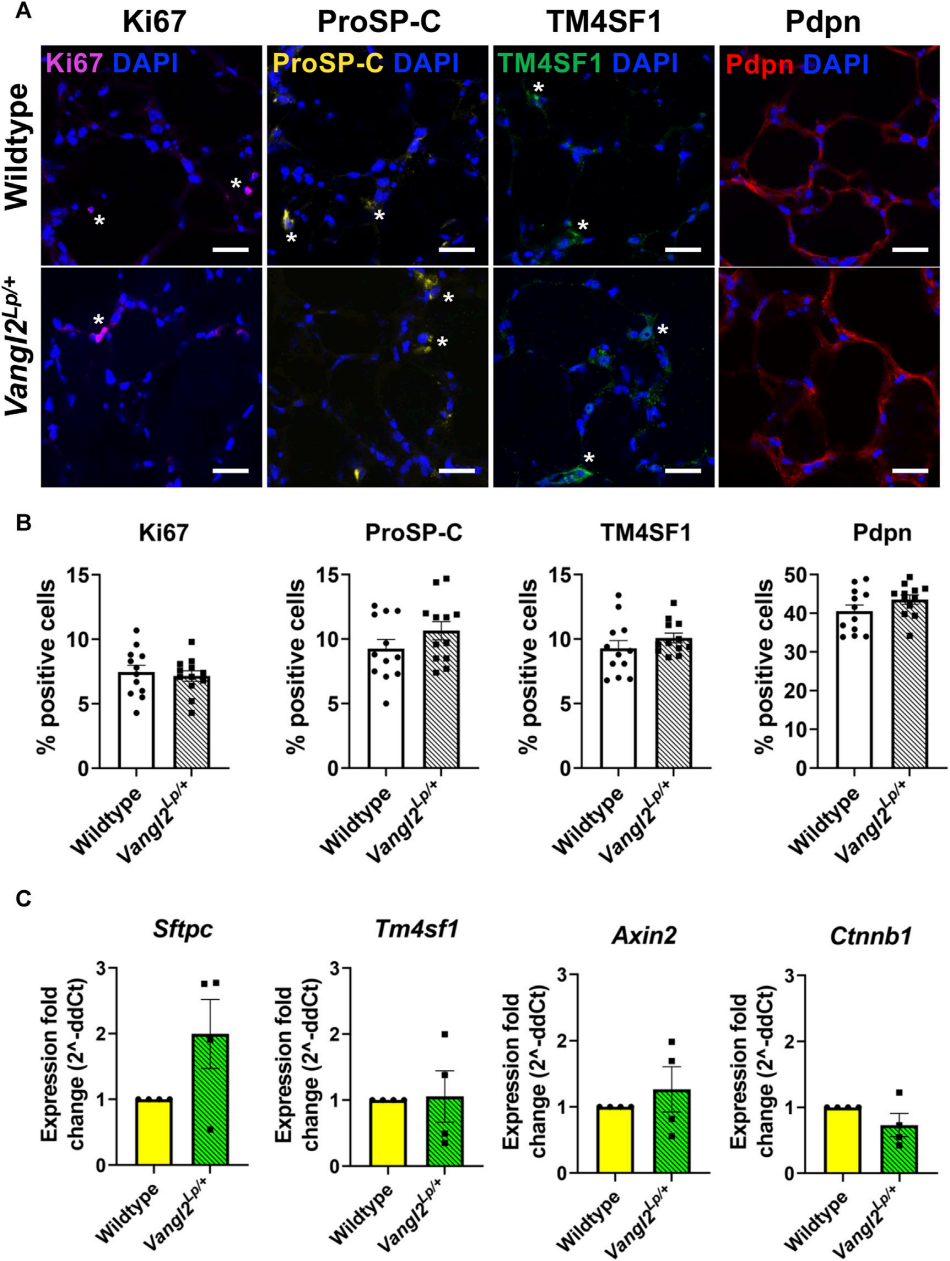
Comparison of key alveolar cell markers in Wildtype and *Vangl2* ^*Lp/+*^ precision-cut lung slices (PCLS). **(A)** Immunofluorescence staining of proliferating cells (Ki67^+^), alveolar type II/progenitor cells (proSP-C^+^), alveolar epithelial progenitor cells (TM4SF1^+^) and alveolar type I cells (Pdpn^+^) in Wildtype and *Vangl2* ^*Lp/+*^ mouse PCLS, scale bars 30 μm (*) highlights examples of cell-type specific staining **(B)** Comparison of the percentage of positive cells in Wildtype and *Vangl2* ^*Lp/+*^ PCLS (each dot represent average value obtained from each PCLS, *n* = 4; Kruskal–Wallis test); **(C)** Relative gene expression fold changes for *Vangl2* ^*Lp/+*^ PCLS normalised to Wildtype PCLS (*n* = 4; Mann Whitney-U test).

We have previously shown a key role for Wnt5a and the planar cell polarity pathway in alveolar homeostasis and repair following injury, suggesting the PCP pathway could be a viable target for regenerative medicine treatments but whether or not PCP signalling affects cell composition is unknown. To investigate whether disruption of the PCP pathway led to changes in the cellular composition of lung tissue, we obtained precision-cut lung slices (PCLS) from *Vangl2* ^*Lp/+*^ and WT mice. PCLS from both genotypes were immunostained using established markers for key epithelial cell populations: alveolar type II (ATII)/progenitor cells (proSP-C^+^); alveolar epithelial progenitor cells (TM4SF1^+^) alveolar type I (ATI) cells (Pdpn^+^) and for proliferating cells (Kim et al., 2021). No statistically significant differences were found in ATII, ATI, alveolar epithelial progenitor cells or proliferating cells between WT and *Vangl2* ^*Lp/+*^ PCLS indicating that the percentage of these cell populations was similar in both WT and *Vangl2* ^*Lp/+*^ PCLS (Figures 1A, B). *Sftpc* and *Tm4sf1* transcript levels were also not statistically different between Wildtype and *Vangl2* ^*Lp/+*^ PCLS, indicating that the gene expression levels were not altered as a result of PCP pathway dysfunction (Figure 1C).

### Canonical Wnt signalling is unaltered in *Vangl2* ^*Lp/+*^ PCLS

Since perturbation of non-canonical Wnt signalling has been shown to alter the level of canonical Wnt signalling and *vice versa* (Rao and Kuhl, 2010; Rogers and Scholpp, 2022), we investigated whether canonical Wnt signalling is altered as a result of PCP pathway dysfunction, by comparing gene expression levels of, *Axin2* and *Ctnnb1,* 2 key canonical pathway genes*.* In agreement with our previous findings from whole, intact *Vangl2* ^*Lp/+*^ lungs (Poobalasingam et al., 2017), *Axin2* and *Ctnnb1* expression levels were not altered in *Vangl2* ^*Lp/+*^ PCLS compared to WT, indicating that dysfunction of the PCP pathway did not alter canonical Wnt signalling in lung slices (Figure 1C).

### Cellular responses to acid injury are similar in WT and *Vangl2* ^*Lp/+*^ PCLS

**FIGURE 2:**
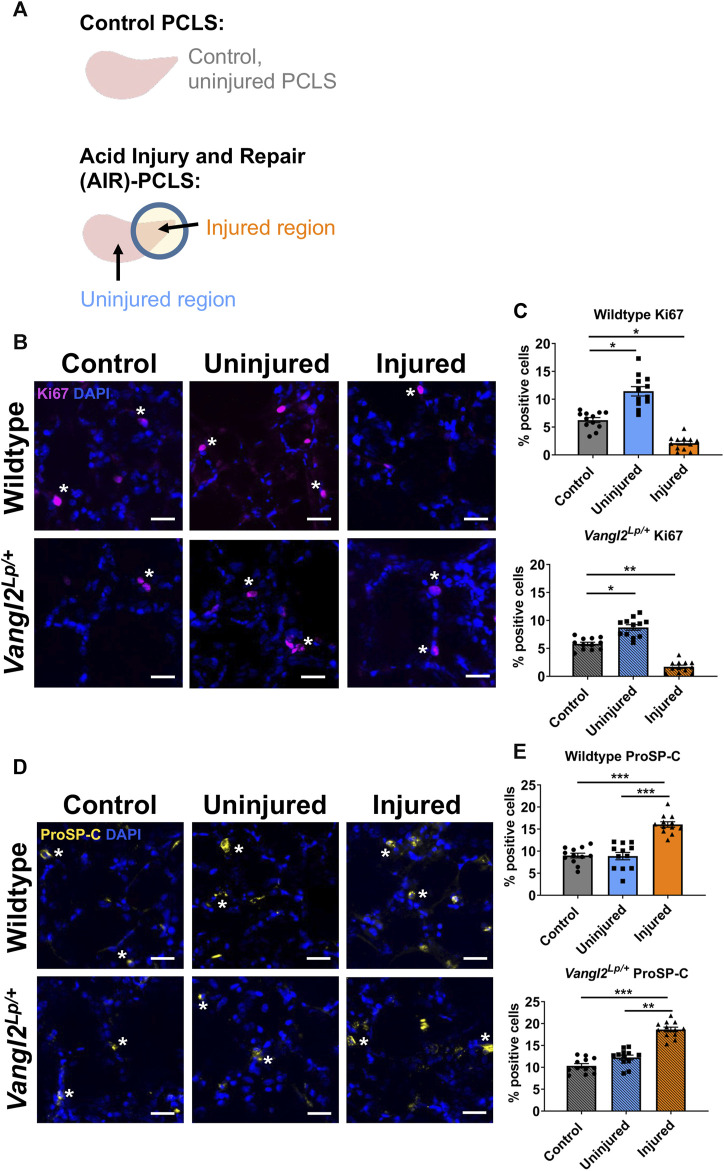
Comparison of cellular responses to injury in Wildtype and *Vangl2* ^*Lp/+*^ PCLS. **(A)** Schematic diagrams of Control PCLS and Acid Repair and Injury (AIR) model in PCLS; Immunofluorescence staining and quantification of Ki67^+^ proliferating cells **(B, C)** and proSP-C^+^ alveolar type II/progenitor cells **(D, E)** in the AIR model in Wildtype and *Vangl2* ^*Lp/+*^ PCLS, scale bars 30 μm (*) highlights examples of cell-type specific staining **(D,E)** (each dot represents average value obtained from each PCLS, *n* = 4; Kruskal–Wallis test, *p < 0.05, **p < 0.01, ***p < 0.001).

In Kim et al. (Kim et al., 2021), we reported that there is an induction of alveolar progenitor cells and a reduction in proliferating cells in injured lung tissue. To compare the repair response of WT and *Vangl2* ^*Lp/+*^ PCLS, we employed the AIR model where hydrochloric acid (HCl) is briefly applied to a spatially restricted region of a lung slice to injure a restricted portion of the PCLS, adjacent to an uninjured area of tissue (Figure 2A). Quantification revealed a decrease in the percentage of proliferating cells (6.2%-2.1%, *p* = 0.014) in the injured region (Figures 2B, C) and a 7.1% increase in proSP-C positive cells (9.0%–16.0%, *p* < 0.001) in the injured region (Figures 2D, E) compared to WT control uninjured PCLS. In *Vangl2* ^*Lp/+*^ PCLS, similar changes in proSP-C (10.3%–18.6%, *p* < 0.001) and Ki67 (5.8%–1.7%, *p* = 0.009) were found (Figures 2B–E). We also saw increased percentages of Ki67 positive cells in the uninjured region compared to control PCLS for both WT (6.2%–11.4%, *p* = 0.028) and *Vangl2* ^*Lp/+*^ (5.8%–8.7%, *p* = 0.041) PCLS (Figures 2B, C). Supplementary Figure S1 shows data from Figures 2C, E plotted on one graph. These data showed that *Vangl2* ^*Lp/+*^ lungs retain the ability to mount a repair response following injury.

### Wnt5a treatment enhances the percentage of alveolar epithelial cells following acid injury

**FIGURE 3:**
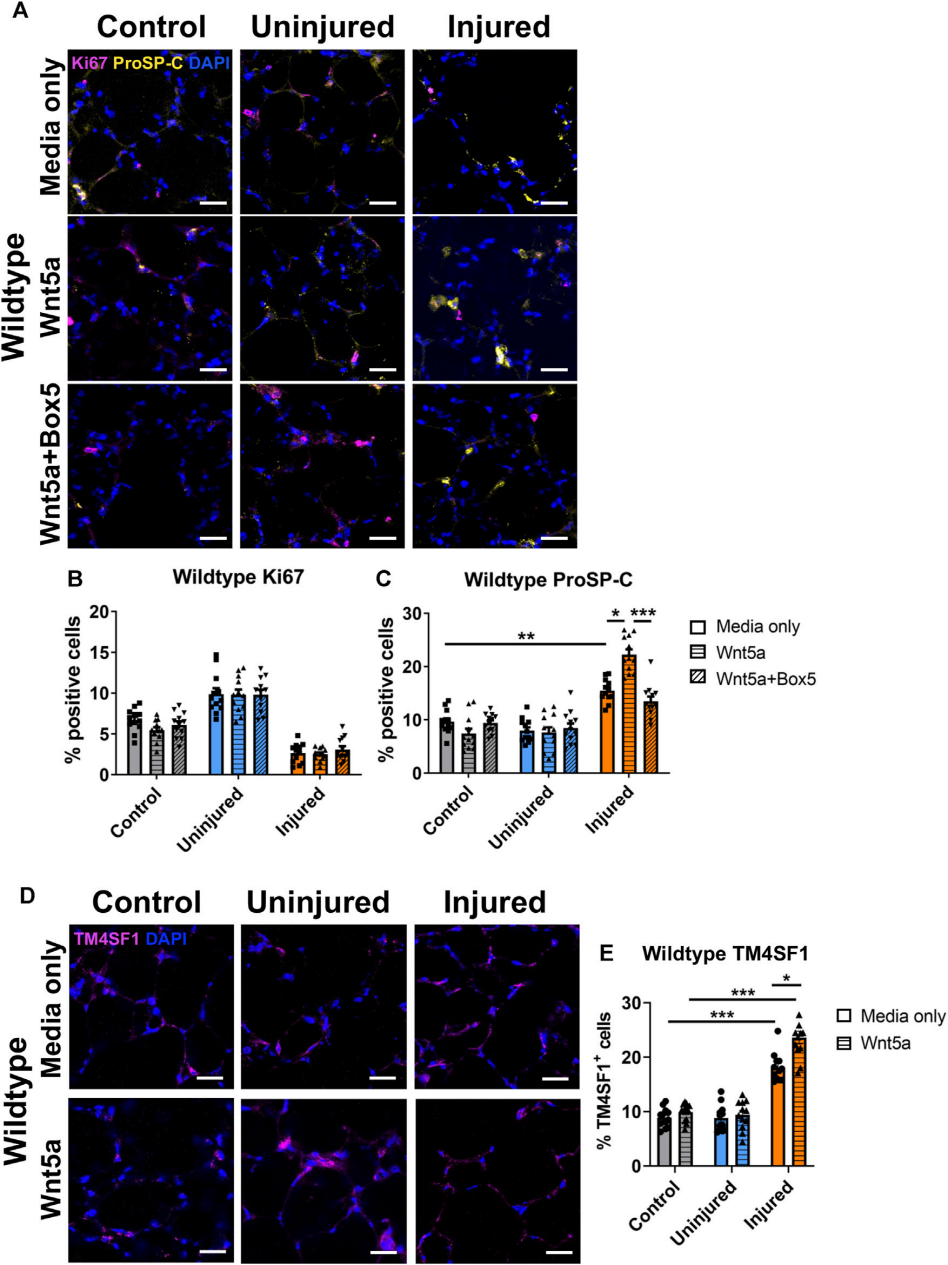
The effects of injury and Wnt5a treatment on progenitor cells and proliferation in Wildtype PCLS. Immunofluorescence staining **(A)** and quantification of Ki67^+^ proliferating cells **(B)** and proSP-C^+^ alveolar type II/progenitor cells **(C)** in the acid injury and repair (AIR) model in Wildtype PCLS upon treatment with Wnt5a alone or Wnt5a and Box5 compared to media only; Immunofluorescence staining **(D)** and quantification of TM4SF1^+^ alveolar epithelial progenitor cells **(E)** in Wildtype AIR-PCLS upon Wnt5a treatment compared to media only; scale bars 30 μm (*n* = 4; Two-way ANOVA, *p < 0.05, **p < 0.01, ***p < 0.001).

Wnt5a, is a key ligand for the Wnt-PCP pathway and this pathway is important for lung repair (Poobalasingam et al., 2017; Cheong et al., 2020). Additionally, Wnt5a has been linked to lung regeneration *in vivo* (Nabhan et al., 2018). To decipher the cellular effects of Wnt-PCP on lung tissue repair, we first looked at the effects of Wnt5a modulation on WT lungs. Wnt5a was applied to WT AIR-PCLS directly after injury and compared to untreated AIR-PCLS. To ensure that the changes were specifically due to Wnt5a, some AIR-PCLS were treated with both Wnt5a and a specific inhibitor of Wnt5a, Box5. In the absence of any injury (WT control uninjured PCLS), neither Wnt5a nor Box5 treatment affected the percentage of Ki67^+^ or proSP-C^+^ cells in WT PCLS (Figures 3A–C).

As reported by Kim et al. (Kim et al., 2021), there was a significant increase in the percentage of proSP-C^+^ cells in the injured region of WT AIR-PCLS compared to control, uninjured PCLS, from 9.7% to 15.5% (*p* = 0.001, Figures 3A, C). Interestingly, treatment with Wnt5a led to a further, statistically significant increase in the percentage of proSP-C^+^ cells in the injured region of WT AIR-PCLS by 6.8% from 15.5% to 22.3% (*p* = 0.011, Figures 3A, C). TM4SF1, is a cell surface marker for alveolar epithelial progenitor cells and approximately 70% of proSP-C^+^ cells co-stain for TM4SF1 (Zacharias et al., 2018). As with proSP-C^+^ cells, the percentage of TM4SF1^+^ cells significantly increased in the injured region of AIR-PCLS compared to control, uninjured PCLS (from 8.9% to 18.1%, *p* < 0.001, Figures 3D, E) (Kim et al., 2021). Treatment with Wnt5a did not alter the percentage of TM4SF1^+^ cells in control, uninjured WT PCLS but as seen with proSP-C, in the injured region of AIR-PCLS, Wnt5a treatment significantly augmented the percentage of TM4SF1^+^ cells by 5.5%, from 18.1% in media only to 23.6% with Wnt5a (*p* = 0.049, Figures 3D, E).

Dual treatment with Wnt5a and its inhibitor, Box5, abolished the additional increase in proSP-C^+^ cells that resulted from Wnt5a treatment back to the levels seen in the injured region of WT AIR-PCLS without any treatment (22.3%–13.5%, *p* = 0.001, Figures 3A, C).

**FIGURE 4:**
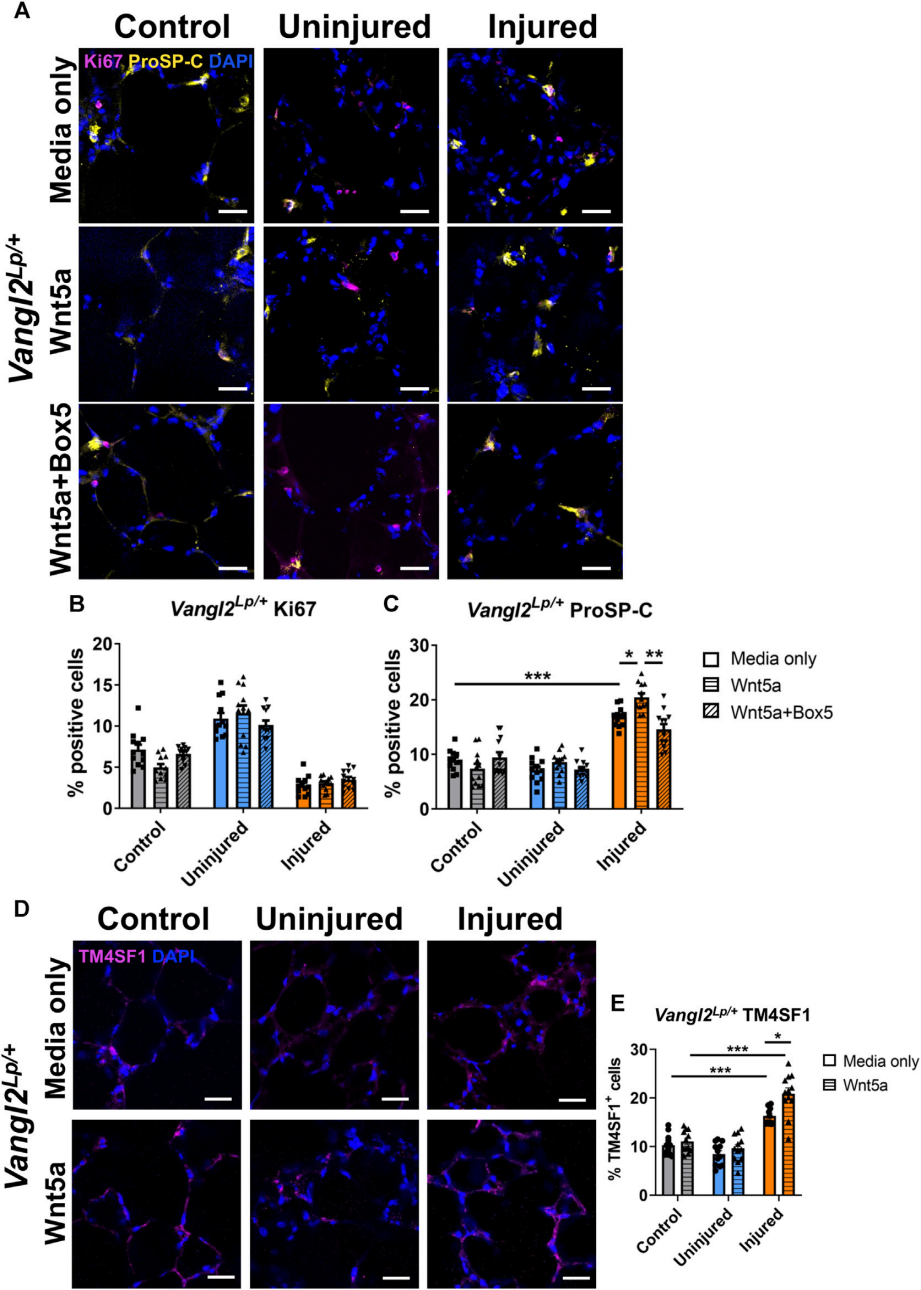
The effects of injury and Wnt5a treatment on progenitor cells and proliferation in *Vangl2* ^*Lp/+*^ PCLS. Immunofluorescence staining **(A)** and quantification of Ki67^+^ proliferating cells **(B)** and proSP-C^+^ alveolar type II/progenitor cells **(C)** in *Vangl2* ^*Lp/+*^ AIR-PCLS upon treatment with Wnt5a alone or Wnt5a and Box5; Immunofluorescence staining **(D)** and quantification of TM4SF1^+^ alveolar epithelial progenitor cells **(E)** in *Vangl2* ^*Lp/+*^ AIR-PCLS treated with Wnt5a or media only; scale bars 30 μm (*n* = 4; Two-way ANOVA, *p < 0.05, **p < 0.01, ***p < 0.001).

In contrast, Wnt5a treatment did not influence cell proliferation. The percentages of Ki67^+^ cells remained unaffected in WT PCLS regardless of Wnt5a/Box5 treatments (Figures 3A, B). Supplementary Figures S2 and S3 show data from WT and *Vangl2* ^*Lp/+*^ shown in Figures 3B, C and Figures 4B, C plotted together.

While the percentage of alveolar epithelial progenitor cells increases in response to injury as part of the innate repair processes, our results showed that exogenous Wnt5a can potentiate the induction of progenitor cells that occurs in response to injury.

### The effects of Wnt5a treatment on alveolar epithelial cells are attenuated, but not abolished in *Vangl2* ^*Lp/+*^ lungs

As seen with WT lung, in the absence of any injury (control uninjured PCLS), neither Wnt5a nor Box5 treatment affected the percentage of Ki67^+^ or proSP-C^+^ cells in *Vangl2* ^*Lp/+*^ PCLS (Figures 4A–C). In untreated *Vangl2* ^*Lp/+*^ AIR-PCLS we still found a significant increase in the percentage of proSP-C^+^ cells (from 8.9% to 16.9%, *p* < 0.001, Figures 4A, C) and TM4SF1^+^ cells (from 10.2% to 16.3%, *p* < 0.001, Figures 4D, E) within the injured region of *Vangl2* ^*Lp/+*^ AIR-PCLS compared to control, uninjured PCLS.

Interestingly, treatment with Wnt5a still resulted in a significant additional increase in the percentage of proSP-C^+^ cells (from 16.9% in media only to 20.5% in Wnt5a, *p* = 0.031, Figures 4A, C) and in TM4SF1^+^ cells (from 16.3% in media only to 20.9% in Wnt5a, *p* = 0.0438, Figures 4D, E), indicating that Wnt5a can still augment the percentage of progenitor cells induced following injury even in the absence of an intact PCP pathway. Though the magnitude of this additional increase in AEP cells was lower than in WT at 3.6% for proSP-C^+^ cells (*p* = 0.031, Figure 4C) and 4.6% for TM4SF1^+^ cells (*p* = 0.044, Figure 4E).

Dual treatment with Wnt5a and its inhibitor, Box5, again abolished the additional increase in percentage of proSP-C^+^ cells back to the levels seen in the injured region of untreated *Vangl2* ^*Lp/+*^ AIR-PCLS (20.5%–14.6%, *p* = 0.003, Figure 4C).

Wnt5a treatment did not influence cell proliferation in *Vangl2* ^*Lp/+*^ PCLS. The percentages of Ki67^+^ cells remained unaffected regardless of Wnt5a/Box5 treatments (Figures 4A, B).

### Neither *Vangl2* dysfunction nor Wnt5a treatment alters the injury induced reduction in ATI cells

**FIGURE 5:**
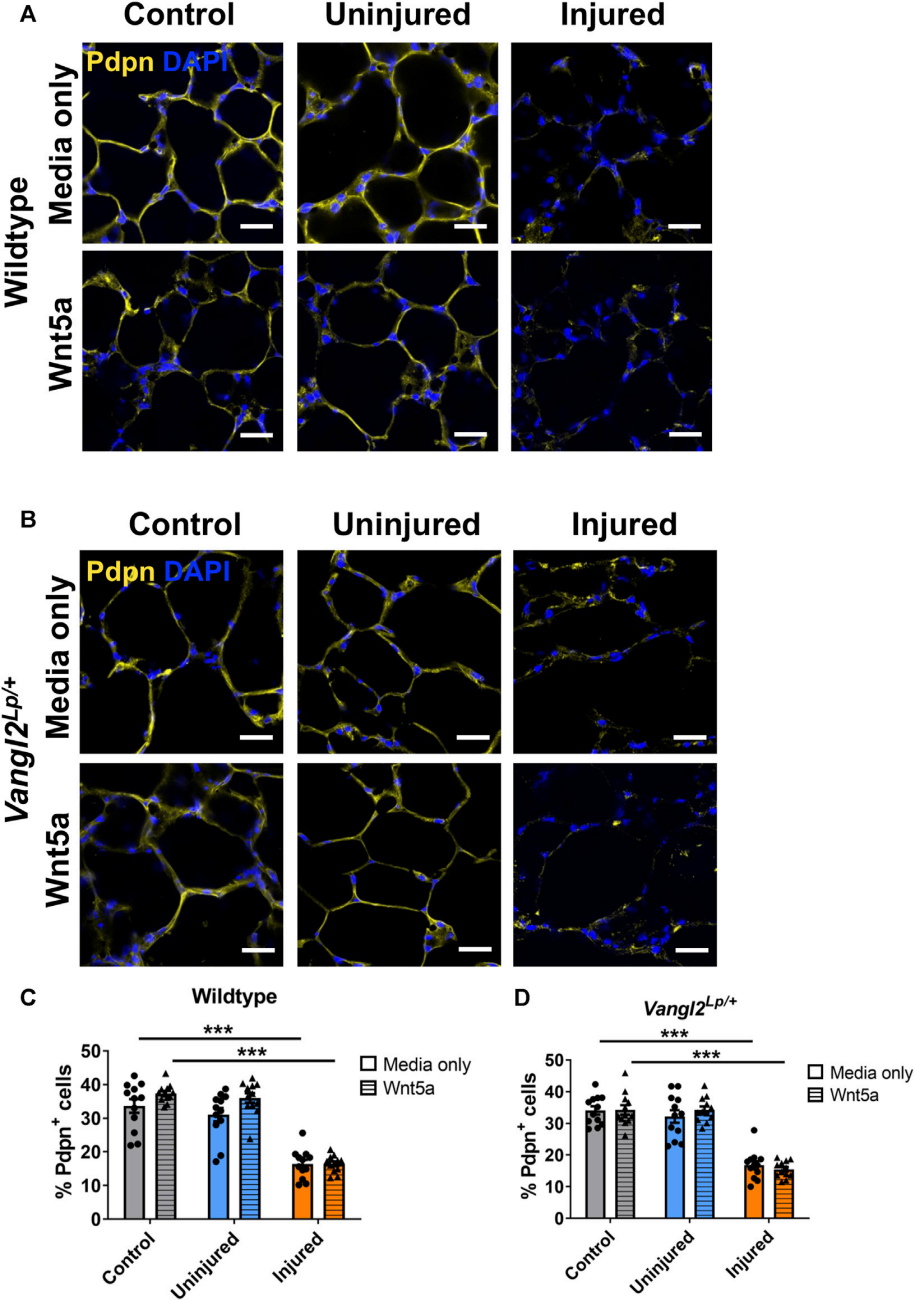
The effects of injury and Wnt5a treatment on alveolar type I cells. Immunofluorescence staining of alveolar type I cells labelled with podoplanin (Pdpn^+^) in Wildtype AIR-PCLS **(A)** or *Vangl2^Lp/+^* PCLS **(B)** treated with media only or media with Wnt5a; Quantification of Pdpn^+^ cells in control PCLS and uninjured and injured regions of Wildtype AIR-PCLS; **(C)** and *Vangl2^Lp/+^* PCLS **(D)** scale bars 30 μm, *n* = 4; Two-way ANOVA, ***p < 0.001.

While induction of ATII derived proSP-C^+^/TM4SF1^+^ progenitor cells is a key response to injury, gathering evidence also indicates that ATI cells exhibit plasticity and can generate ATII cells in order to restore damaged epithelium (Penkala et al., 2021). We therefore investigated the response of ATI cells to Wnt5a and Vangl2 dysfunction in AIR PCLS by quantifying cells immunostained for the ATI marker podoplanin (Pdpn). The percentage of Pdpn^+^ ATI epithelial cells was significantly reduced after injury, this was consistent in both WT (from 33.7% to 16.3%, *p* < 0.001) and *Vangl2* ^*Lp/+*^ (from 34.1% to 16.8%, *p* < 0.001) PCLS (Figures 5A–D). Wnt5a treatment did not affect the percentage of Pdpn^+^ cells in control, uninjured PCLS for either WT (from 33.7% to 37.4%, *p* = 0.657, Figures 5A, C) or *Vangl2* ^*Lp/+*^ (from 34.1% to 34.2%, *p* > 0.999, Figures 5B, D). There was also no change in the percentage of Pdpn^+^ cells in uninjured or injured regions of AIR-PCLS upon Wnt5a treatment for either WT (from 31.0% to 36.0% in uninjured, *p* = 0.073, and from 16.3% to 16.4% in injured, *p* > 0.999, Figures 5A, C) or *Vangl2* ^*Lp/+*^ (from 32.2% to 34.2% in uninjured, *p* = 0.884, and from 16.8% to 15.5% in injured, *p* = 0.958, Figures 5B, D) PCLS.

### Acid injury leads to an increase in vimentin^+^ staining in *Vangl2* ^*Lp/+*^ PCLS

**FIGURE 6:**
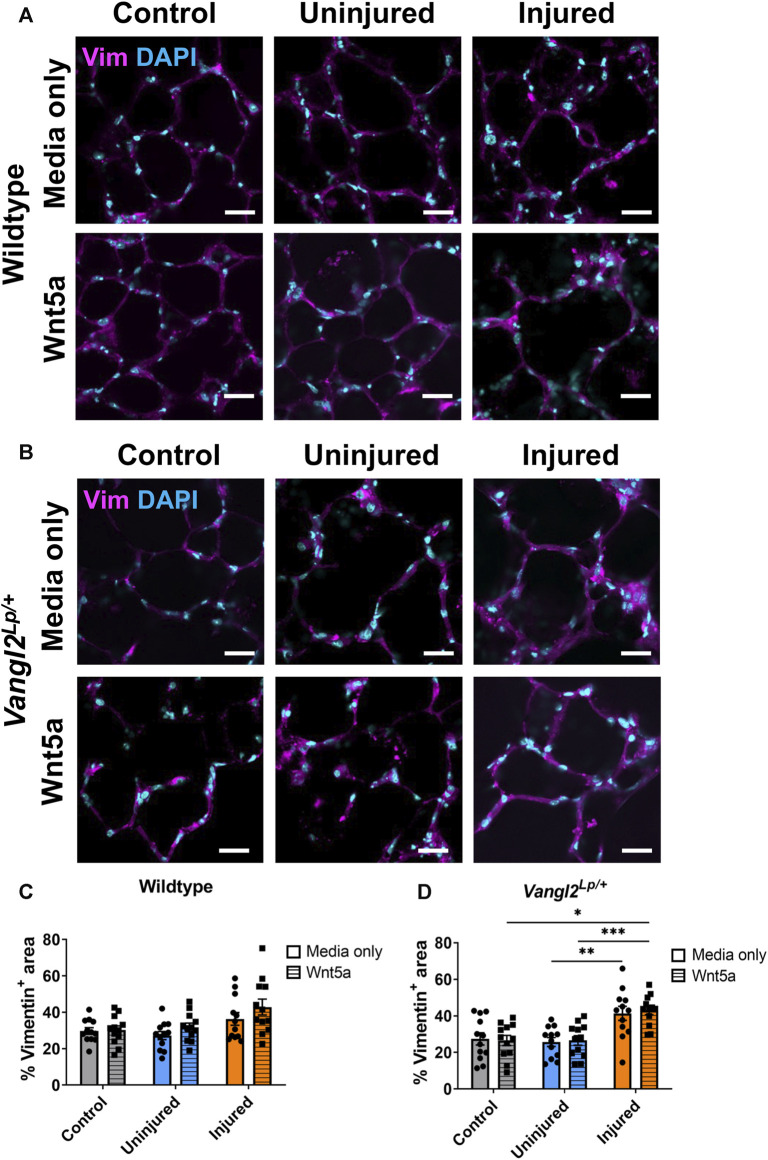
The effects of injury and Wnt5a treatment on fibroblasts. Immunofluorescence staining of fibroblasts labelled with vimentin (Vim) antibody in Wildtype **(A)** or *Vangl2^Lp/+^* **(B)** AIR-PCLS treated with Wnt5a or media; **(C)** Quantification of Vim^+^ area as a percentage of whole tissue area in Wildtype control PCLS and in uninjured and injured regions of AIR-PCLS with Wnt5a treatment or media only; **(D)** Quantification of Vim^+^ area as a percentage of whole tissue area in *Vangl2* ^*Lp/+*^ control PCLS and in uninjured and injured regions of *Vangl2* ^*Lp/+*^ AIR-PCLS with Wnt5a treatment or media only; Scale bars 30 μm, *n* = 4, Two-way ANOVA, *p < 0.05, **p < 0.01, ***p < 0.001.

It has been shown that following influenza-induced lung injury *in vivo*, Wnt5a is secreted from fibroblasts to stimulate induction of epithelial progenitor cells (Nabhan et al., 2018). Additionally, during repair, cells including fibroblasts, may migrate, proliferate and/or spread to re-cover denuded areas of tissue (Crosby and Waters, 2010). To investigate whether Wnt5a treatment or PCP pathway dysfunction affected fibroblasts following injury, we immunostained WT and *Vangl2* ^*Lp/+*^ control or AIR-PCLS cultured with or without Wnt5a for the fibroblast marker vimentin (Figures 6A, B). Quantification of vimentin^+^ cells is challenging due to the widespread distribution of this protein throughout the cytoplasm and the varied morphology of different fibroblast sub-types. Therefore, we developed a custom-made macro to quantify the percentage area covered by fibroblasts within the PCLS to capture potential fibroblast responses (Figures 6C, D).

In WT PCLS, although there was an increase in Vimentin^+^ area in the injured region of AIR-PCLS compared to either control PCLS (from 29.7% to 36.3%, *p* = 0.407) or uninjured regions of AIR-PCLS (from 27.2% to 36.3%, *p* = 0.172), this was not statistically significant. There was also no significant change in vimentin^+^ area of injured regions of AIR-PCLS compared to either control PCLS (from 30.2% to 42.9%, *p* = 0.230) or uninjured regions of AIR-PCLS (from 31.8% to 42.9%, *p* = 0.112) following Wnt5a treatment (Figures 6A, C).

However, in *Vangl2* ^*Lp/+*^ there was a significant increase between uninjured and injured regions of AIR-PCLS (from 25.7% to 41.3%, *p* = 0.003, Figures 6B, D). Interestingly, Wnt5a treatment also led to a significant increase in vimentin^+^ area in the injured region of *Vangl2* ^*Lp/+*^ AIR-PCLS compared to both control *Vangl2* ^*Lp/+*^ PCLS (from 26.5% to 43.0%, *p* = 0.011, Figures 6B, D) and the uninjured region of *Vangl2* ^*Lp/+*^ AIR-PCLS (from 26.5% to 43.0%, *p* = 0.001).

### *Vangl2* ^*Lp/+*^ alveolar epithelial cells show diminished capacity to migrate towards a chemoattractant

**FIGURE 7:**
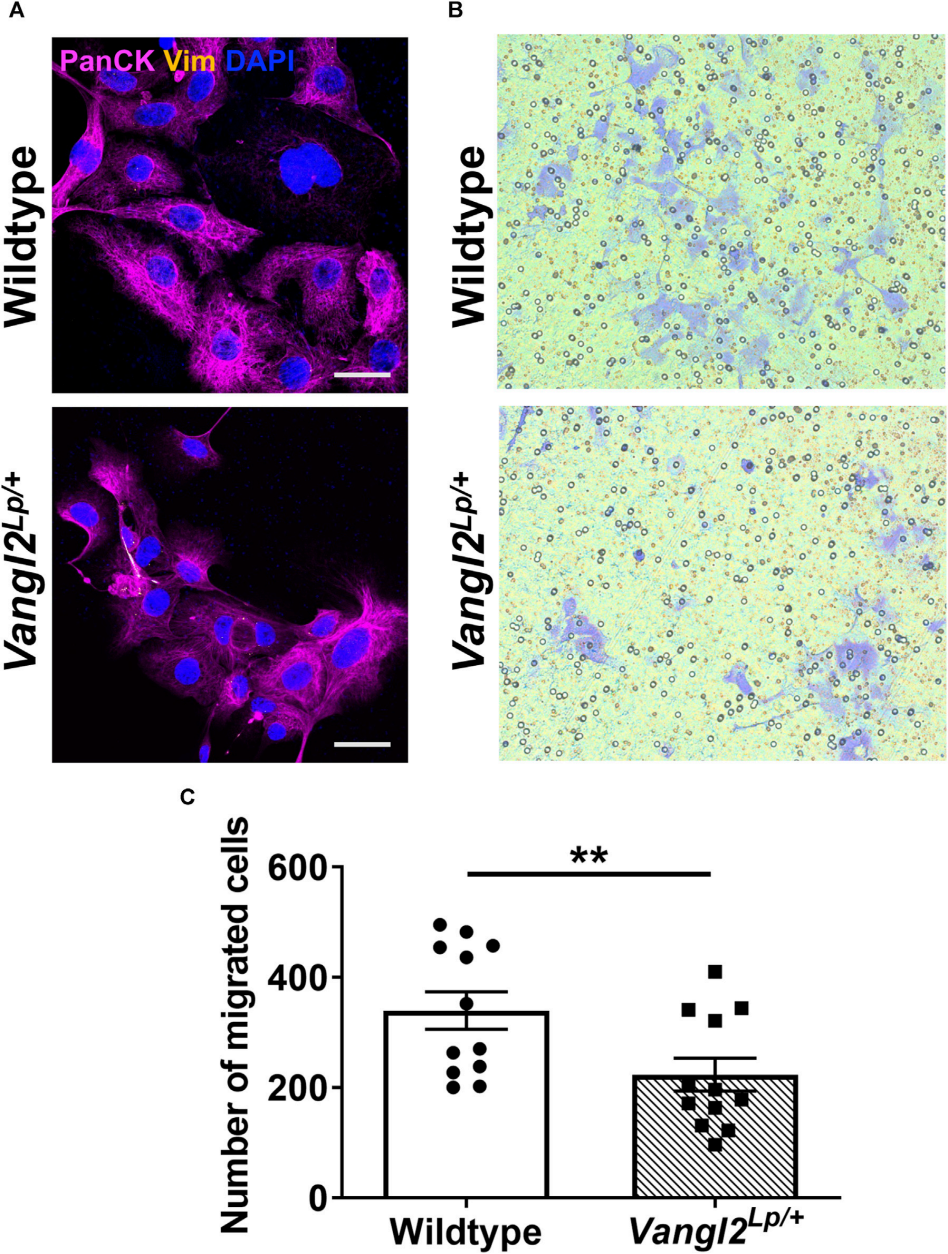
Alveolar epithelial cells isolated from *Vangl2* ^*Lp/+*^ lungs have reduced migratory capacity. **(A)** Immunofluorescence staining with pan-cytokeratin (epithelial marker) and vimentin (fibroblasts) confirms the identity of epithelial cells isolated from Wildtype and *Vangl2* ^*Lp/+*^ lungs, imaged using confocal microscopy, scale bars 30 μm; **(B)** Images of migrated cells stained with crystal violet, following Transwell migration assays; **(C)** Quantification of the number of migrated cells in five fields of view following Transwell migration assay (*n* = 4; Mann-Whitney *U* test, **p < 0.01).

We have previously shown that tracheal epithelial cells (TECs) isolated from *Vangl2* ^*Lp/+*^ mice have a reduced capacity to migrate following a scratch injury and live imaging of post-natal day 3 *Vangl2* ^*Lp/+*^ epithelial cells revealed disrupted cell migration resulting in impaired alveolar formation (Cheong et al., 2020). To determine whether disruption of the Wnt-PCP pathway would affect migration of adult alveolar epithelial cells (AECs) towards a chemoattractant, we conducted transwell migration assays. AECs were isolated from WT and *Vangl2^Lp/+^* lungs using magnetic bead-based cell separation. CD31^neg^, CD45^neg^ and CD326 (EpCAM)pos cells were isolated. Epithelial cell identity was confirmed by positive staining for pan-cytokeratin and negative staining for vimentin (Figure 7A). In addition, we confirmed that at 24h post-isolation, almost all the isolated cells were positive for the alveolar epithelial cell markers proSP-C and/or podoplanin (Supplementary Figure S4). Cells were seeded onto 8 μm pore Transwell inserts at 100,000 cells per well in DMEM/F12 medium supplemented with 5% fetal bovine serum (FBS) in the apical compartment. Media in the basal compartment was supplemented with 10% FBS and fibroblast growth factor-10 (FGF10) to induce migration. AECs were cultured for 48h before staining with crystal violet solution and quantification. The cells that did not migrate were removed prior to quantification of cells that migrated through the pores (Figure 7B). There was a 34% reduction in the number of *Vangl2^Lp/+^* AECs that had migrated through the pores compared to WT (mean = 223 cells per transwell for *Vangl2^Lp/+^* and 340 cells per transwell for WT, n = 4, *p* = 0.008, Figure 7C). This data showed that, migration towards a chemoattractant is impaired in AECs from adult *Vangl2^Lp/+^* mice.

### Atomic force microscopy reveals altered cell stiffness profiles in *Vangl2* ^*Lp/+*^ tracheal epithelial cells

**FIGURE 8:**
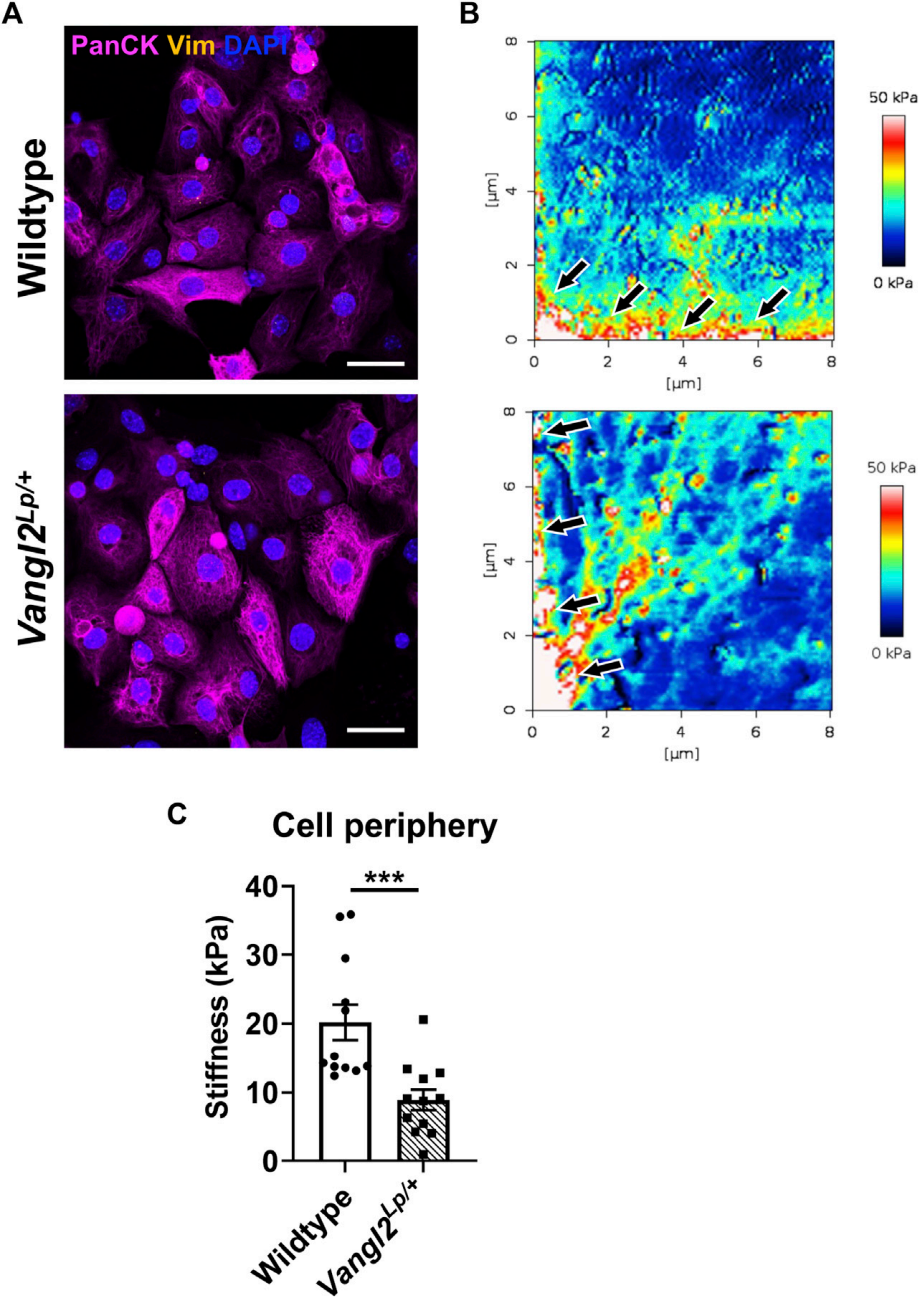
Epithelial cells isolated from Wildtype and *Vangl2* ^*Lp/+*^ trachea have different mechanical properties. **(A)** Immunofluorescence staining of epithelial cells (pan-cytokeratin) and fibroblasts (vimentin) confirms the identity of epithelial cells isolated from trachea of Wildtype and *Vangl2* ^*Lp/+*^ mice, imaged using confocal microscopy, scale bars 30 μm; **(B)** Comparison of Young’s modulus (stiffness) micrographs obtained using atomic force microscopy, arrows indicate cell periphery where stiffness was quantified; **(C)** Comparison of stiffness in the cell periphery of Wildtype and *Vangl2* ^*Lp/+*^ epithelial cells (*n* = 4; Mann-Whitney *U* test, ***p < 0.001).

*Vangl2* ^*Lp/+*^ TECs exhibit reduced migration following injury as a result of disruption to the actomyosin network and impaired traction forces (Cheong et al., 2020). This prompted us to assess whether disruption of the planar cell polarity pathway might also affect cell stiffness. To do this, we isolated TECs from WT and *Vangl2* ^*Lp/+*^ mice as previously described (Cheong et al., 2020). Epithelial cell identity was confirmed by immunostaining for the epithelial marker cytokeratin and absence of the fibroblast marker, vimentin (Figure 8A). The measurement of cell stiffness at nanoscale levels, using atomic force microscopy revealed that stiffness measured at the cell periphery was significantly lower in *Vangl2* ^*Lp/+*^ TECs (Figure 8B). WT TECs exhibited a mean stiffness of 20.2 kPa whereas stiffness of *Vangl2* ^*Lp/+*^ TECs was more than 55% lower at 8.9 kPa (Figure 8C, *p* < 0.001).

## Discussion

### Non-canonical Wnt/PCP pathway in lung repair

Wnt5a is critical for most stages of lung development, including the saccular and alveolar stages (Li et al., 2005; Loscertales et al., 2008; Sucre et al., 2020; Li et al., 2022). In addition, multiple studies have highlighted the clinical relevance of Wnt5a in adult and congenital lung diseases (Baarsma et al., 2017; Martin-Medina et al., 2018; Yuan et al., 2019; Sucre et al., 2020). Lack of Wnt5a results in alveolar insufficiency and modulation of this protein is being actively pursued as a potential treatment for bronchopulmonary dysplasia, a disease of prematurity where alveologenesis is impaired (Li et al., 2020; Sucre et al., 2020; Ai et al., 2022).

Both Wnt5a and many of the PCP pathway genes are critical for *in utero* development and therefore most homozygous mutants do not survive post-natally. Consequently, most studies involving genetic modification of Wnt-PCP pathway genes have been limited to embryonic stages of life. Even mice with conditional deletion of *Wnt5a*, *Ror2* or *Vangl2* in either the distal lung epithelium or mesenchyme only did not survive beyond the first week after birth, precluding analysis of the Wnt-PCP pathway in adult lungs (Li et al., 2008; Zhang et al., 2020).

Using the AIR model, we have investigated the precise cellular effects of Wnt5a and the PCP pathway on early stages of alveolar lung repair in complex adult lung tissue. Our data reveals that Wnt5a specifically enhances the generation of AEP cells following lung injury but does not affect either ATI cells at this stage of repair.

Notably, we observed an increase in fibroblast coverage in *Vangl2* ^*Lp/+*^ mice only, which was significant after acid injury in both untreated (media only) Wnt5a treated AIR-PCLS. *In vivo* Wnt5a is thought to be secreted by fibroblasts to stimulate adjacent progenitor cells in response to injury (Nabhan et al., 2018), so it was surprising that we did not see a similar response in WT lung tissue. However, our analysis only measured one aspect of fibroblast biology (cell size/spreading) at 48h post injury, further analysis, such as quantification of fibroblast sub-types or analysis of additional time-points would provide a more comprehensive picture of the effects of the Wnt-PCP pathway on this cell type during repair.

### Wnt5a and PCP- one pathway or more?

Whilst we found a specific increase in AEP following Wnt5a treatment. Surprisingly in *Vangl2* ^*Lp/+*^ PCLS where the PCP pathway is disrupted, Wnt5a still led to an increase in AEP following injury, although the increase was attenuated compared to that seen in WT alveoli. This result shows that Wnt5a is still capable of exerting pro-repair effects in the absence of an intact PCP pathway in agreement with our previous findings that Wnt5a can still stimulate wound healing in *Vangl2* ^*Lp/+*^ tracheal epithelial cells (Cheong et al., 2020). The underlying mechanism through which Wnt5a orchestrates alveolar repair in the absence of VANGL2, is still unclear. In agreement with our previous analysis of whole lung, we did not see a difference in the canonical pathway gene β-catenin or the canonical signalling reporter gene Axin2 in lung slices. Though, we cannot rule out that there could be differences in specific cell populations within the slices which are masked by analysing whole tissue slices. The current study, in conjunction with our previous findings (Cheong et al., 2020), provides evidence that an alternative factor is present that can transmit Wnt5a induced signalling to drive repair when VANGL2 function is compromised. Given the strikingly similar phenotypes observed in alveolar development and repair among *Wnt5a*, *Vangl2*, and *Ror2* mouse mutants, the receptor tyrosine kinase Ror2 is a promising candidate. Ror2 possesses an extracellular cysteine-rich domain (CRD) that can bind directly to Wnt5a and previous studies have shown that Wnt5a/ROR activates downstream small GTPases, as well as the c-Jun N-terminal kinase (JNK) pathway, to modulate cell polarisation and migration, critical biological processes in the context of tissue repair (Oishi et al., 2003; Nomachi et al., 2008; Yu et al., 2016). Further studies will be required to precisely elucidate the role of Ror2 in governing Wnt5a-mediated repair following lung injury.

Interestingly a recent study in Zebrafish showed that rather than operating in one pathway, PCP genes and Wnt ligands work in parallel to establish proper mechanosensory hair cell orientation (Navajas Acedo et al., 2019), it is therefore possible that Wnt5a and the PCP pathway co-operate in repair but through separate pathways.

### The Wnt-PCP pathway and lung repair

Harnessing Wnt signalling for therapeutic approaches is challenging because of the complexity and limited pathway specific ligands and receptors. Moreover, different cellular outcomes can result, depending on which pathway is stimulated, for example, proliferation, differentiation, migration (Rao and Kuhl, 2010). Modulating the Wnt5a-PCP pathway could provide a viable approach to regenerate and repair damaged tissue and treat diseases through modification of the actin cytoskeleton, without some of the potentially damaging effects that targeting the canonical Wnt pathway might bring such as altering proliferation.

Recent work on the causes of PAH has highlighted the potential of targeting the Wnt-PCP pathway to treat this disease (Goncharova et al., 2023). The Wnt5a-PCP pathway is required to maintain pericyte coverage of pulmonary microvessels, thereby helping to support the small vessels in the pulmonary vasculature. Loss of these small vessels is a hallmark of PAH. Yuan et al. demonstrated that mice with an endothelial specific deletion in Wnt5a have persistent PAH and importantly, treatment of pulmonary microvascular endothelial cells obtained from PAH patients with Wnt5a enriched exosomes enhanced the migration/repair effects in wound-healing assays (Yuan et al., 2019).

### PCP dysfunction and cell stiffness

Through its role in regulating the actin cytoskeleton, previous studies have shown that the PCP pathway is important for cell migration and mechanosignalling. Since biophysical cues can regulate stem cell behaviour and fate (Titushkin et al., 2010), it was important for us to not only look at the effects of PCP disruption on specific alveolar sub-types but also to investigate any changes in biophysical parameters.

Here we investigated the migratory capacity of primary alveolar epithelial cells in response to a chemoattractant, to mimic the *in vivo* molecular cues that epithelial cells might receive following tissue injury. Significantly fewer *Vangl2* ^*Lp*^ mutant cells migrated towards a chemoattractant source compared to WT cells showing that migratory capacity is reduced upon PCP pathway disruption. Because of the role of *Vangl2* in mechanosignalling (Cheong et al., 2020), we also looked at the effect of PCP pathway dysfunction on tracheal epithelial cell stiffness and found there was a significant difference at the cell periphery, where actin cytoskeleton and cortical actin regulate cell stiffness. The significantly lower stiffness of TECs in the cell periphery aligned with our previous findings that *Vangl2* ^*Lp/+*^ TECs have disrupted focal adhesions around the cell periphery that generate significantly less traction forces (Cheong et al., 2020).

## Conclusion

This study provides important information about the cellular mechanisms through which Wnt5a and the PCP pathway contribute to lung alveolar repair. We show that Wnt5a plays a key role by enhancing the induction of alveolar epithelial progenitor cells following injury, suggesting that this growth factor could be developed for use as a pro-repair treatment for the lungs. We have focused here on deciphering the early repair responses, up to 48h after injury. In future, it will be important to understand the longer-term consequences of Wnt5a and PCP manipulation on lung repair. It is likely that to optimise tissue repair, whilst avoiding detrimental side effects, pro-repair treatments may need to be combined with bioengineering approaches, for example encapsulation, to spatially and/or temporally regulate the duration of therapeutic effects and target them to specific regions of tissue damage.

## Data Availability

The original contributions presented in the study are included in the article/Supplementary Material, further inquiries can be directed to the corresponding author.
