# Supplementary figures and images for: Deciphering the impacts of modulating the Wnt-planar cell polarity (PCP) pathway on alveolar repair
Source: Front Cell Dev Biol. 2024 Feb 27;12:1349312. doi: 10.3389/fcell.2024.1349312 (PMC10927798; doi:10.3389/fcell.2024.1349312)

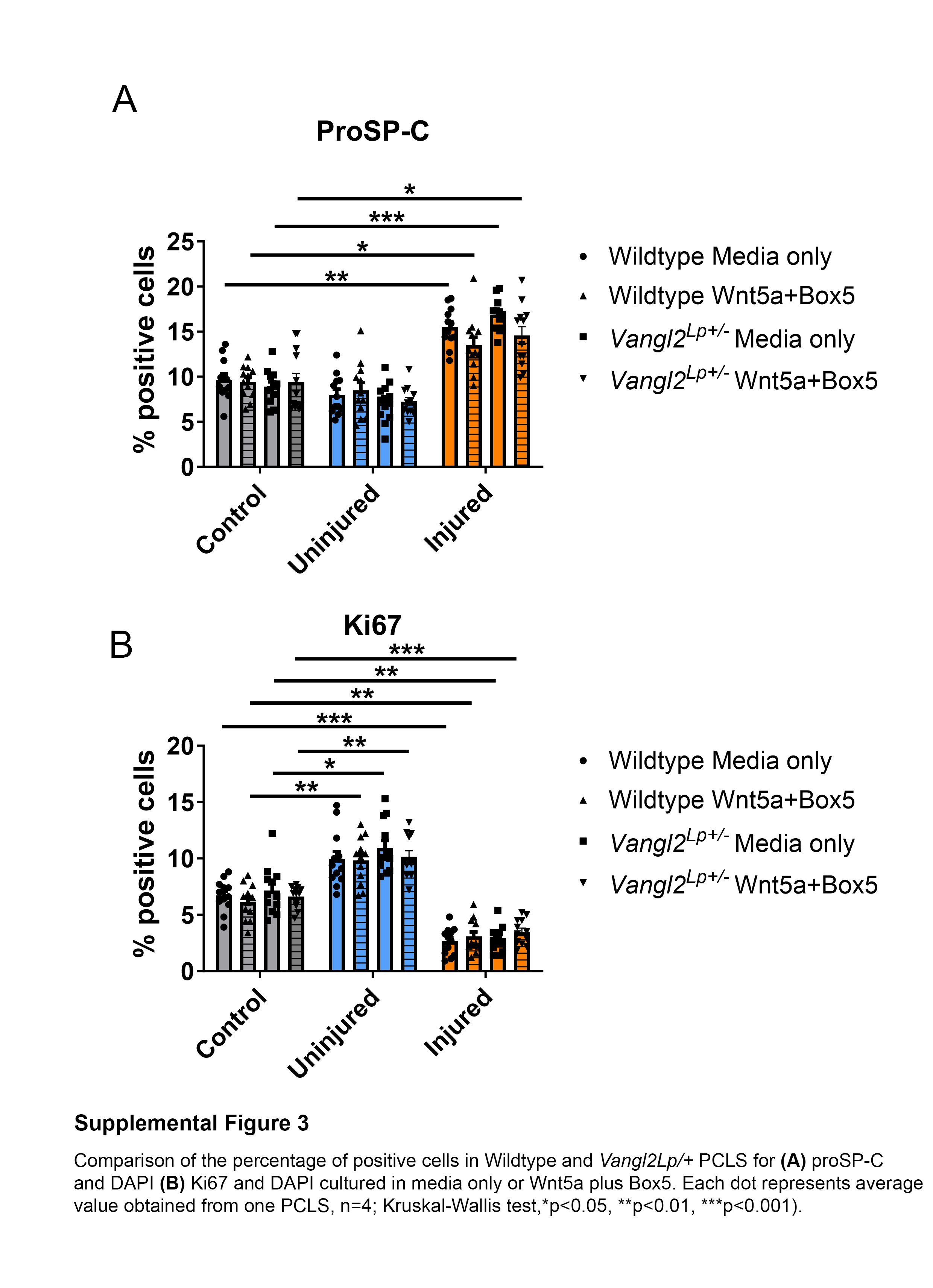

Supplement: Supplementary file 1 [file Image3.TIF]

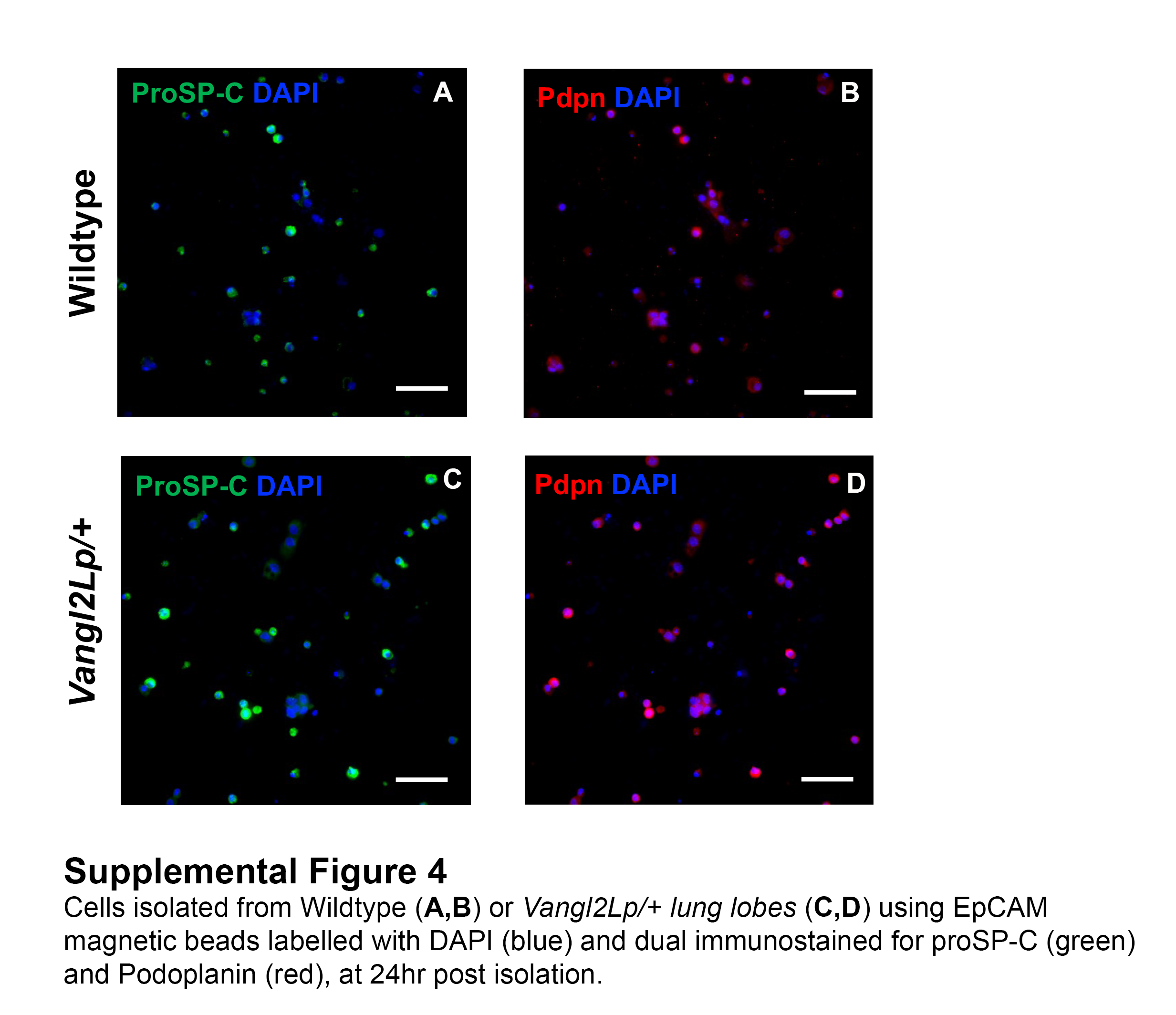

Supplement: Supplementary file 2 [file Image4.TIF]

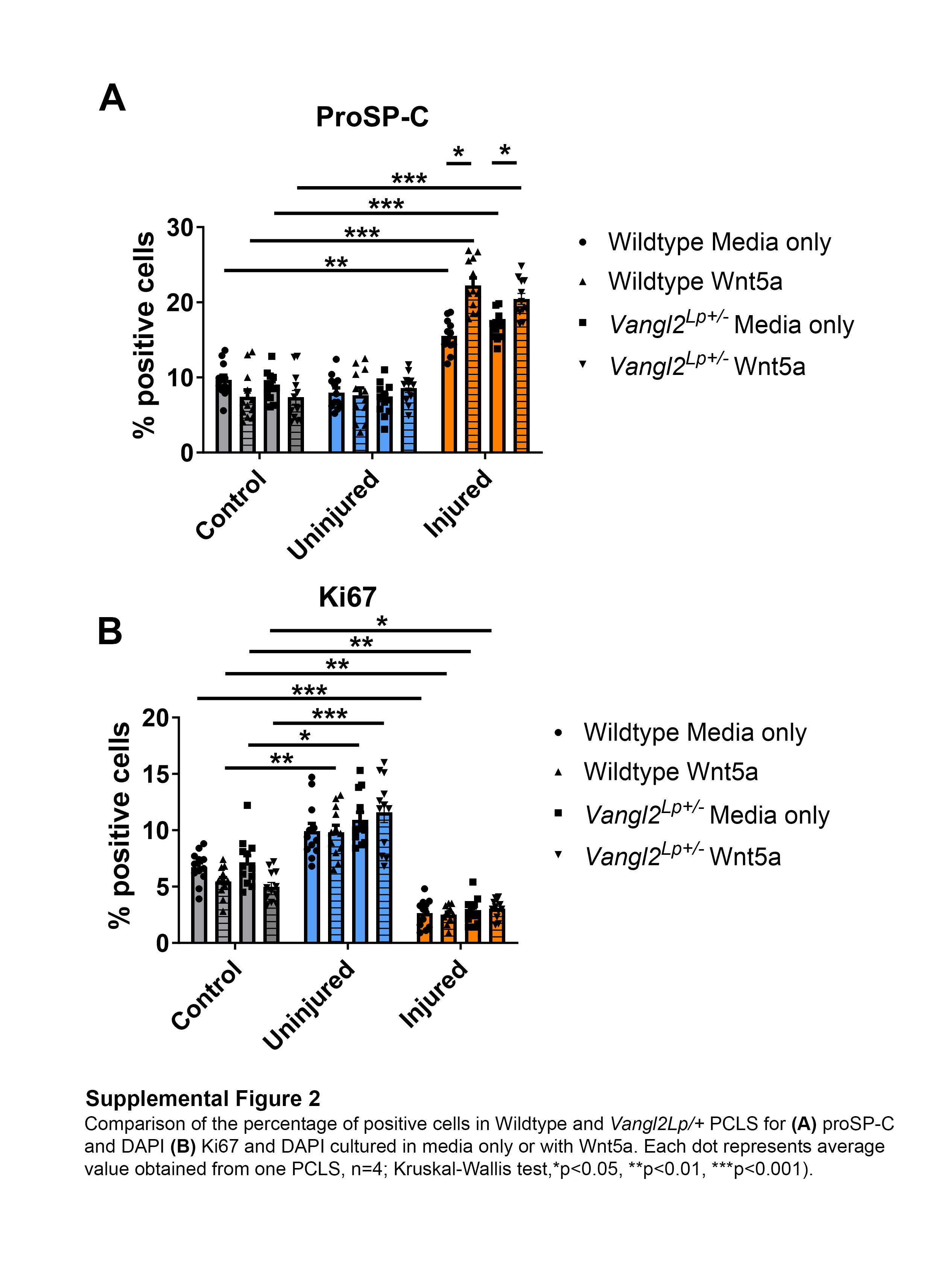

Supplement: Supplementary file 3 [file Image2.TIF]

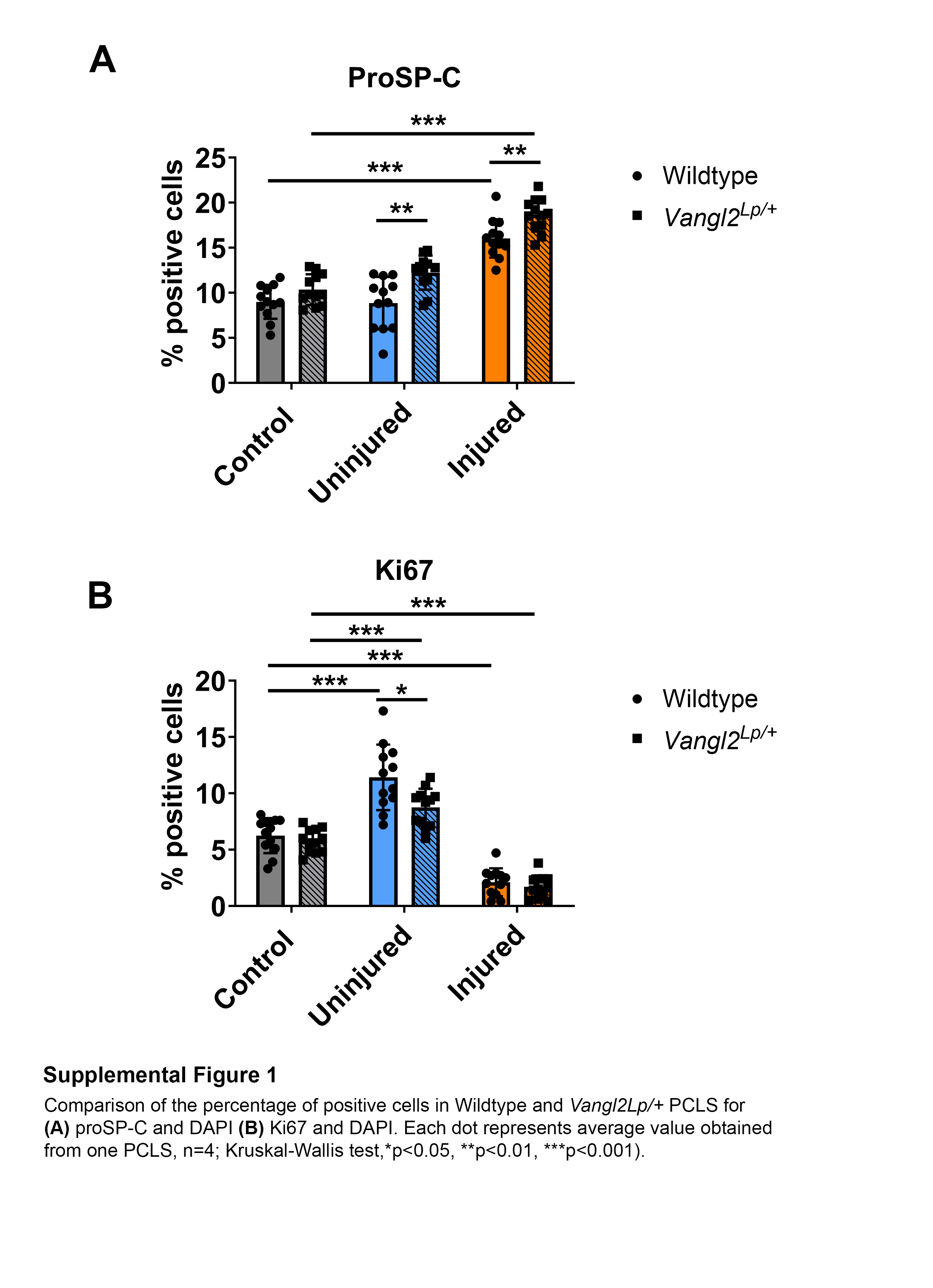

Supplement: Supplementary file 4 [file Image1.TIF]
